# Series-elastic actuator with two degree-of-freedom PID control improves torque control in a powered knee exoskeleton

**DOI:** 10.1017/wtc.2023.20

**Published:** 2023-10-16

**Authors:** Sergei V. Sarkisian, Lukas Gabert, Tommaso Lenzi

**Affiliations:** 1 Department of Mechanical Engineering and the Utah Robotics Center at the University of Utah, Salt Lake City, UT, USA; 2 Rocky Mountain Center for Occupational and Environmental Health, Salt Lake City, UT, USA

**Keywords:** powered exoskeletons, wearable robotics, series elastic actuators, rehabilitation robotics

## Abstract

Powered exoskeletons need actuators that are lightweight, compact, and efficient while allowing for accurate torque control. To satisfy these requirements, researchers have proposed using series elastic actuators (SEAs). SEAs use a spring in series with rotary or linear actuators. The spring compliance, in conjunction with an appropriate control scheme, improves torque control, efficiency, output impedance, and disturbance rejection. However, springs add weight to the actuator and complexity to the control, which may have negative effects on the performance of the powered exoskeleton. Therefore, there is an unmet need for new SEA designs that are lighter and more efficient than available systems, as well as for control strategies that push the performance of SEA-based exoskeletons without requiring complex modeling and tuning. This article presents the design, development, and testing of a novel SEA with high force density for powered exoskeletons, as well as the use of a two degree-of-freedom (2DOF) PID system to improve output impedance and disturbance rejection. Benchtop testing results show reduced output impedance and damping values when using a 2DOF PID controller as compared to a 1DOF PID controller. Human experiments with three able-bodied subjects (*N* = 3) show improved torque tracking with reduced root-mean-square error by 45.2% and reduced peak error by 49.8% when using a 2DOF PID controller. Furthermore, EMG data shows a reduction in peak EMG value when using the exoskeleton in assistive mode compared to the exoskeleton operating in transparent mode.

## 1. Introduction

Powered exoskeletons are wearable robotic devices that can amplify the user’s movements during ambulation and functional mobility. The applications of powered exoskeletons include rehabilitation (Cempini et al., 2013), assistance (Sarkisian et al., 2020), and strength amplification (Kazerooni, 2005). Powered exoskeletons have been rapidly evolving from tethered devices designed for basic research in the laboratory (Veneman et al., 2007; Celebi et al., 2013; Zhang et al., 2015; Witte et al., 2017) to fully autonomous systems that promise to improve the user’s quality of life at home and in the community (Ishmael et al., 2021; Slade et al., 2022). Many open challenges remain to achieve the full potential of powered exoskeletons (Yan et al., 2015). Despite continuous progress, there is still an unmet need for lightweight, efficient electromechanical actuators that can effectively convert electrical energy into mechanical power to assist the user’s joint.

Series elastic actuators (SEA) are commonly used in powered exoskeletons. There are several advantages of using SEAs compared to more commonly used fully rigid actuators. First, the elastic element of the SEA combined with a position sensor can act as a force sensor, allowing for fast and precise force measurement (Williamson and Pratt, 1995; Paluska and Herr, 2006; Veneman et al., 2007). Second, the elastic element of the SEA reduces motor speed requirements by passively storing and releasing mechanical energy, resulting in potential electrical and mechanical energy savings (Au and Herr, 2008; Au et al., 2009; Tran et al., 2022). Additionally, the elasticity of the actuator acts as a mechanical low-pass filter and improves the actuator’s impact load resistance. However, these benefits come at the cost of increased actuator mass, size, and mechanical complexity. Therefore, there is a need for new design solutions that can decrease the weight and complexity of SEAs.

A multitude of SEA designs for powered lower-limb exoskeletons has been proposed. A common implementation of SEAs for powered exoskeletons consists of using a torsional spring in combination with planetary or harmonic-drive gearboxes (Chen et al., 2017; Kim and Bae, 2017; Aguirre-Ollinger and Yu, 2021; Zhang et al., 2022). These solutions tend to be quite compact but have some drawbacks. Planetary gearboxes with high transmission ratios suffer from low efficiency and poor backdrivability. Moreover, planar torsional springs are difficult to design with the range of stiffness and torque necessary for powered exoskeletons, often resulting in heavy, custom steel springs with a large diameter in relation to the electrical motor and gearbox.

Another common approach for the design of SEAs consists of using linear springs in combination with a linkage system, creating a torsional spring. This approach allows using lighter, off-the-shelf springs with high energy density. However, it requires using multiple opposed compression coil springs and swingarms to emulate a bidirectional torsional spring (Kwa et al., 2009; Karavas et al., 2012; Zhu et al., 2014; Qian et al., 2022). Leaf springs made of composite materials like fiberglass have also been used in SEAs (Meijneke et al., 2014; Shepherd and Rouse, 2017). Although composite springs can store a large amount of energy per unit mass, they are highly nonlinear and pose challenges with deformation measurement as well as long-term life due to delamination. Linear springs can be combined with both rotary and linear actuators, with the latter typically resulting in the highest torque and power density (Pratt and Krupp, 2004; Dollar and Herr, 2008; Paine et al., 2014; Sarkisian et al., 2020; Ishmael et al., 2022).

High-performance torque control is often necessary for powered exoskeletons. SEAs simplify the torque control problem by providing a reliable and accurate measure of the output torque through the spring deformation (Pratt et al., 2002). To obtain high performance of torque control, researchers often use PID regulators in combination with friction compensation (Banala et al., n.d.). This approach is simple to implement but does not fully account for the intrinsic dynamics of the actuation system. Therefore, its performance deteriorates noticeably under highly dynamic tasks, which require quick changes in the direction of the output joint. Inertia compensation has been demonstrated to address this issue, although it is harder to accomplish, as it requires carefully selected filters and gains to guarantee stability (Aguirre-Ollinger et al., 2011). Another control approach leading to high performance in highly dynamic tasks consists of using a disturbance observer (DOB) (Paine et al., 2014). However, DOBs are difficult to implement because they require an accurate dynamic model of the system as well as carefully selected filters to avoid distortions of the output torque and instability. Thus, the development of simple, high-performance controllers for SEA-driven exoskeletons is an open question.

In this article, we present a novel SEA with high force density combined with a two degree-of-freedom (2DOF) PID controller to provide accurate torque control in highly dynamic tasks for powered exoskeletons. To achieve high force density, we used a coil spring and a capturing system which enabled the emulation of a bidirectional torsional spring using only one coil spring for both compression and extension. Moreover, we used a ball screw system with a high transmission ratio, high mechanical efficiency, and high backdrivability. To improve the performance of the torque controller, we implemented a 2DOF PID controller (Taguchi and Araki, 2000; Roy et al., 2021), which does not require a dynamic model of the actuator or the assisted user joint and can be easily tuned. Rigorous benchtop experiments were conducted to assess the performance of the proposed actuator and control strategy. Furthermore, we performed human experiments with three able-bodied subjects to validate the proposed SEA design and 2DOF PID control strategy in an assistive use case involving a powered knee exoskeleton. The main contributions of this article are (1) the novel SEA design featuring a single coil spring and a linear actuator to achieve among the highest torque density in the field and (2) the first demonstration of a 2DOF PID controller improving torque control during highly dynamic human–robot interaction tasks.

## 2. Design

**Figure 1.**
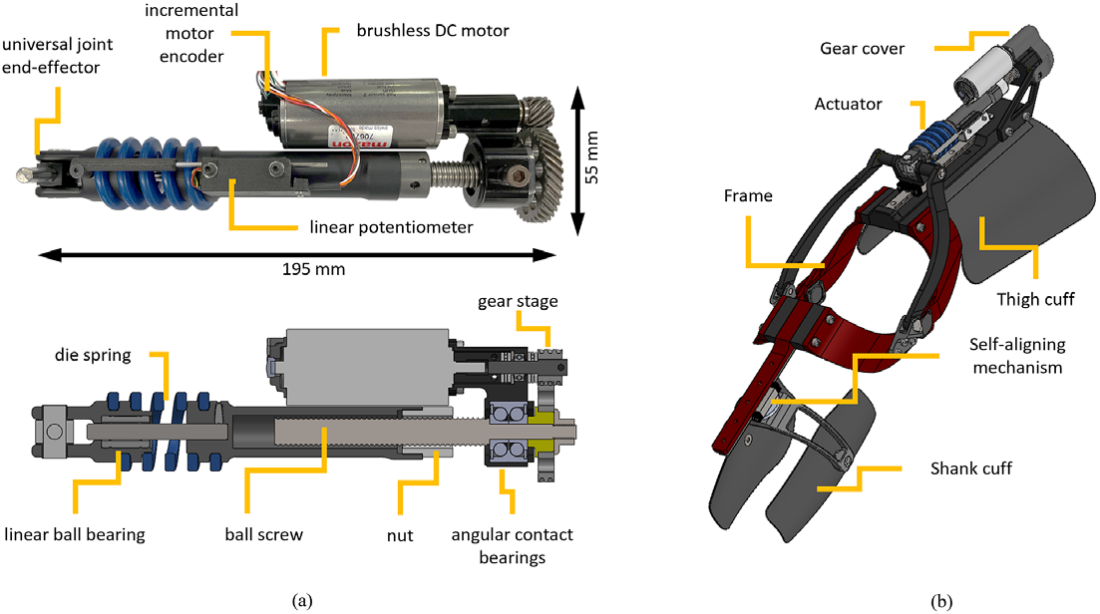
(a) The prototype of the series elastic actuator. The actuator uses a brushless DC motor, a 3:1 gear stage, a 2-mm lead ball screw system, and a die spring. The spring deflection is measured by a linear potentiometer combined with a custom ADC board. (b) Utah ExoKnee powered exoskeleton with the proposed actuator.

We designed a linear SEA with high torque and power density based on a custom spring arrangement that enables a compression coil spring to work both in compression and extension. The custom spring arrangement is connected to a ball-screw and motor combination, as shown in Figure 1, which shows a photo of the actuator prototype and a sectioned view of the actuator model. The actuator uses a brushless DC motor (EC 4-Pole 30 200 W, Maxon Motors, Switzerland) which is connected to a helical drive gear (H2412R, Boston Gear, Charlotte, NC) using a flexible aluminum shaft coupling to eliminate radial forces due to shaft misalignments. The motor-drive gear mates with a larger helical gear (H2436L, Boston Gear, Charlotte, NC), creating a 3:1 gear ratio. The larger gear is mechanically connected using a key to a ball screw (SD 12X2R G7SHAFT-A, Ewellix, Sweden). The ball screw is supported by a double-row angular contact bearing (SRD 10300, Myonic, Chatsworth, CA).

The nut of the ball screw system is threaded to a custom machined part that connects the nut to a coil spring (1.5″ OD X 0.75″ ID Chrome-Silicon Steel Die Spring, McMaster-Carr, Elmhurst, IL), which was modified to have open ends. The machined part connecting the ball-screw nut to the spring features a thread that matches the pitch and diameter of the coil spring and ensures a very tight press fit with no backlash. The other end of the spring is connected to the end-effector of the actuator using a similar custom part featuring a thread that matches the pitch and diameter of the coil spring. Due to this connection mechanism, the spring has a different number of active coils in compression and extension, resulting in different effective lengths. During compressive loading, the ends of the spring interact with the machined parts, effectively reducing the number of active coils by a quarter-coil on each side of the spring. In contrast, during tension loading, the inactive coils are pulled away from the machined parts increasing the effective length of the spring. Due to these differences in effective spring length, we expect the spring assembly to have higher stiffness in compression than extension. The end-effector of the actuator features a universal joint with low-friction polymer bushings (iglide Z, IGUS, Germany) to reduce undesired off-axis loading. A cylindrical linear ball bearing guide is used to ensure coaxiality of the machined parts interfacing with the coil spring regardless of the spring status.

An analog linear potentiometer (10 kΩ ±15%, LMC8–11, P3 America, Leander, TX) measures the spring deflection through a custom analog-to-digital converter (ADC) board. The ADC has an 18-bit resolution (ADS8887IDRCT, Texas Instruments, Dallas, TX) and is located close to the potentiometer to minimize noise. The custom ADC board is 30 mm long and 10 mm wide. The body of the potentiometer and the ADC board are attached to the nut side of the spring, and the shaft of the potentiometer is attached to the end effector side of the spring using a 3D-printed interface. An incremental magnetic rotary encoder measures the motor position and velocity (12-bit, RM08ID, RLS, Slovenia). The actuator is powered by a custom 10-cell Lithium–Ion battery pack (40 T 21700 4,000 mAh 35A battery cells, Samsung SDI, South Korea). The nominal battery voltage is 36 V.

The proposed SEA uses a custom electrical control system. The motherboard features a PIC32 microcontroller (PIC32MK0512MCF100, Microchip Technology Inc., Chandler, AZ) that reads all the sensors and communicates with the current motor driver (G-TWIR 80/80 SES, Elmo Motion Control Ltd., Israel) and the ADC board. The motherboard also features a Raspberry Pi (Compute Module 3+, Raspberry Pi Foundation, Cambridge, England, UK) running high-level code and communicating with the PIC32 using a serial peripheral interface (SPI). The control electronics and the battery pack are enclosed in a 3D-printed box that is worn by the subject at the waist. The box is 135 × 100 × 45 mm and weighs 312 g.

To test the performance of the proposed SEA, we modified the Utah ExoKnee (Figure 1b), a powered knee exoskeleton previously used for physical human–robot interaction studies (Sarkisian et al., 2020, 2021) and stroke assistance (Sarkisian et al., 2022). The frame of the exoskeleton remained largely unchanged, aside from some machined parts to accommodate the specific dimensions of the proposed SEA.

## 3. Control

The low-level controller on the exoskeleton uses a PID algorithm to execute the desired torque received from the high-level controller. Designing a feedback controller that performs well in both set-point tracking and disturbance rejection can be challenging. Achieving fast load disturbance rejection usually requires a controller with high gains, which results in an oscillatory set-point step response. One way to address this issue is by implementing a 2DOF PID controller that combines feedforward and feedback control to improve tracking performance. This can be achieved by assigning weights to the reference signal for the proportional and derivative actions. The degree of freedom of the controller refers to the number of independently tunable transfer functions.

**Figure 2.**
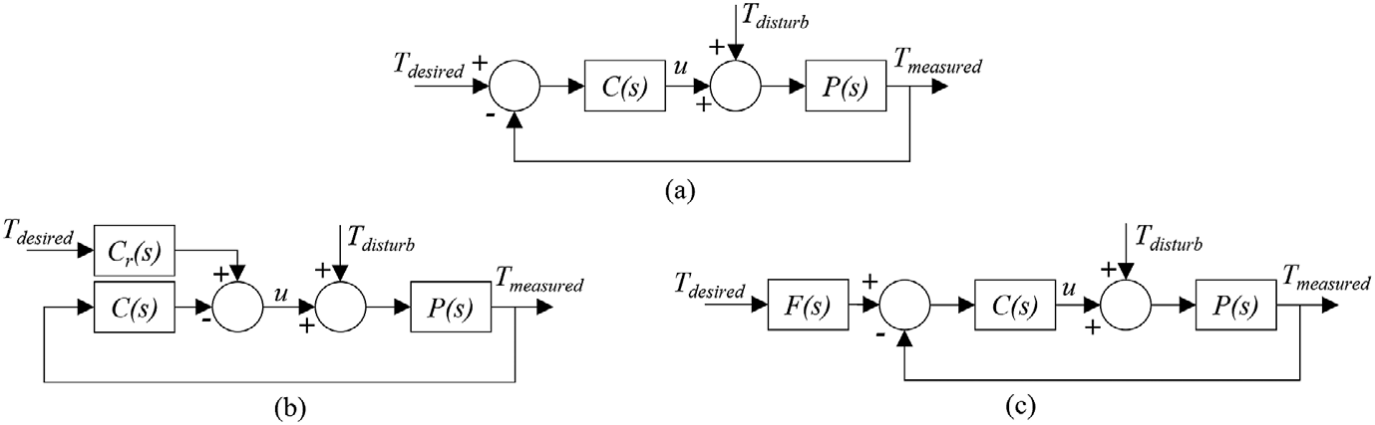
(a) Conventional 1DOF PID low-level controller block diagram. (b) The 2DOF PID low-level controller diagram. (c) The equivalent block diagram of the 2DOF PID controller.

There are two PID algorithms implemented in this study – 1DOF PID (Figure 2a) and 2DOF PID (Figure 2b,c). Although the implementation of a 1DOF PID algorithm is very common, we show the algorithm here for clarity in comparison to the 2DOF PID algorithm. In the 1DOF PID torque controller (Figure 2a) *C(s)* is a PID compensator, *P(s)* is the plant, *T*_desired_ is the reference signal, *T*_measured_ is the measured output, *T_disturb_* is the disturbance, *K_P_, K_I_,* and *K_D_* are the proportional, integral, and derivative gains, respectively.
(1)

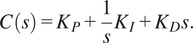

Equation (1) then results in the following control law when combined with the reference *r* and measured output *y.*

(2)

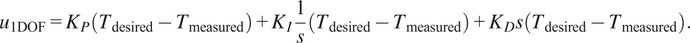



With a 2DOF PID controller, we introduce an additional compensator *C_r_(s)* shown in (2), where the two additional gains *b* and *c* are introduced in association with the *K_P_* and *K_D_* gains.
(3)

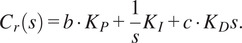



A control law can then be derived for the 2DOF PID system (Figure 2b) using *C(s)* and *C_r_(s).*

(4)

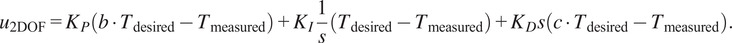



The diagram shown in Figure 2b can be rearranged into an equivalent diagram, as shown in Figure 2c with unity feedback and a transfer function *F(s)* that is derived from *C(s)* and *C_r_(s).*

(5)

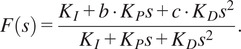



Here, the gains *b* and *c* act as a filter of the change in the reference signal. The values of *b* and *c* can range between 0 and 1. The *b* and *c* gains of the 2DOF PID controller help reject disturbances that naturally occur due to human interaction. Unlike disturbance observers (Paine et al., 2014), a 2DOF PID controller can reject disturbances without requiring a plant model.

## 4. Benchtop experiments

**Figure 3.**
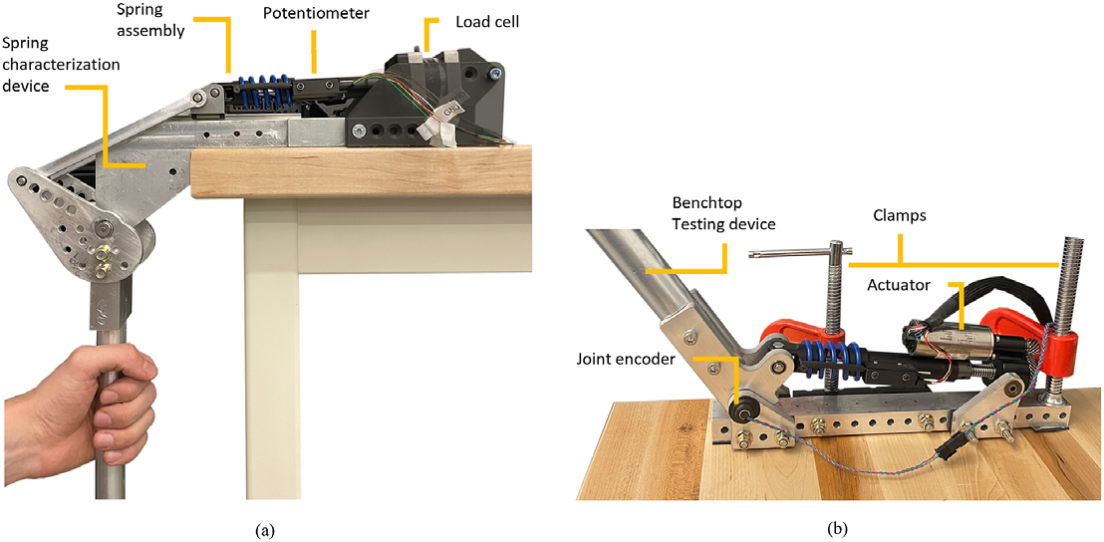
(a) Spring characterization setup. A benchtop device was used to manually drive the output and deform the spring. The spring was connected to a 6-axis load cell in series to measure the applied force. Additionally, a linear potentiometer was used to measure spring deformation. (b) Benchtop testing device used for benchtop actuator and controller characterization. In both cases, the testing devices were firmly clamped to a bench.

We performed benchtop experiments with the SEA spring assembly to characterize the compression and extension stiffness. For this experiment, we used a custom benchtop setting (Figure 3a), which consisted of a four-bar linkage to convert rotary movement of the crank into linear motion of the slider. The crank had a 30 cm handle attached to it for manual backdriving. The slider, supported by a linear guide, was attached to the end-effector side of the spring assembly. The other end of the spring assembly was rigidly attached to a grounded 6-axis load cell (M3713D, Sunrise Instruments, China). The deformation of the spring was measured by the same linear potentiometer described in the previous section.

**Figure 4.**
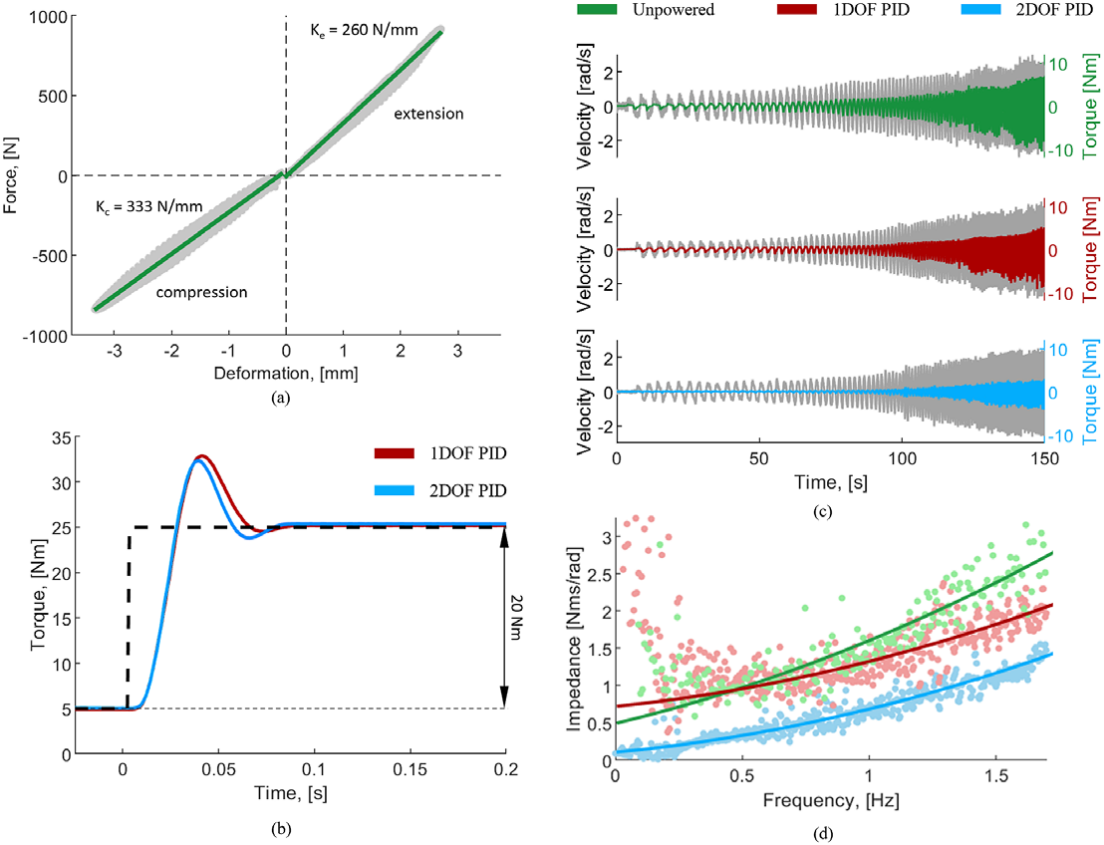
(a) Spring characterization data. The compression and extension stiffnesses were estimated by fitting a line to the load cell and deformation data and estimating the slope of the line. (b) Step response of the low-level controllers. A 5 Nm preload torque was used to eliminate backlash. The desired torque of 20 Nm was used. (c) Actuator backdriving torque during unpowered and controlled conditions (1DOF PID and 2DOF PID). (d) Estimated output impedance as a function of input frequency.

Using the handle, we slowly and progressively loaded and unloaded the SEA spring assembly while we recorded the spring deflection and the loadcell output. Separate trials were performed for tension and compression. We estimated the compression and extension stiffnesses offline by fitting the compression and extension load cell and deformation data separately with two first-order polynomial functions. The estimated spring stiffness values were 333 and 260 N/mm for compression and extension, respectively (Figure 4a). The fitting showed high linearity (*R* ^2^ = 0.996 in compression, *R* ^2^ = 0.989 in extension) and low error (root-mean-square [RMS] error = 1.07% in compression, RMS error = 2.05% in extension). Thus, the experiments show that the spring has linear behavior and that the stiffness is about 22% higher in compression than in extension, as expected from the design.

We characterized the performance of the closed-loop controller using a slightly different benchtop testing device (Figure 3b). This benchtop device worked as an inverted slider-crank four-bar linkage having a 90° range of motion, and sturdy mechanical end stops to ensure safety. The benchtop device used an absolute rotary encoder to measure the angular position of the joint (Linear voltage, RM08Vx, RLS, Slovenia). Similar to the previous test setup, this setup featured a 30 cm long handle attached to the crank of the four-bar linkage for manual backdriving.

Using this custom benchtop setup, we assessed the response of both the 1DOF PID and 2DOF PID controllers to a step change in set point. To this end, we rigidly constrained the output joint using a clamp. Then, we performed 10 repetitions of a 20-Nm torque step input with a pre-load of 5 Nm. We tuned the 2DOF PID and 1DOF PID controllers manually to achieve the same rise time and −3 dB bandwidth. For both PID controllers, we first set all the controller gains to zero. Then, we gradually increased the value of KP until sustained oscillations occur. We then increased the derivative gain KD to dampen the system and reduce the overshoot to below 40%, which is a relatively common value in the literature. Finally, we increased the integral gain KI to minimize steady-state error. For the 2DOF PID controller, we set *b* and *c* values together with KP and KD values, obtaining similar proportional and derivative actions to the 1DOF PID controller, which resulted in the same rise time and bandwidth. The gains of the 1DOF PID controller were selected as follows: *P* = 1.5, *I* = 20, *D* = 0.05. The gains of the 2DOF PID controller were selected as *P* = 2, *I* = 20, *D* = 0.0667, *b* = 0.75, and *c* = 0.75. The offline analysis of the 1DOF PID (Figure 4b) showed an average rise time of 11.9 ms, which corresponds to a −3 dB bandwidth of 29.4 Hz (first-order approximation). Moreover, the average overshoot was 39.5%, the settling time was 79.3 ms, and the steady-state error was 0.166 Nm. Similarly, the offline analysis of the 2DOF PID (Figure 4b) showed an average rise time of 11.9 ms, which corresponds to a −3 dB bandwidth of 29.4 Hz, the average overshoot was 36.5%, the settling time was 76.3 ms, and the steady-state error was 0.371 Nm. Except for some small but visible differences in settling time, the step responses for the 1DOF PID and 2DOF PID were almost identical.

We characterized the output impedance of the proposed SEA by backdriving the actuator manually. For consistency, the experimenter followed a sine sweep, which started at 0.25 Hz and ramped up to 2 Hz over a 150-second period, using both acoustic and visual cueing (Figure 4c). First, the backdriving test was performed while the actuator was unpowered. Then, we repeated the experiment with the actuator powered and set to transparent mode (i.e., zero desired torque) using the 1DOF PID and the 2DOF PID (Figure 4c).

We used the recorded data to estimate the maximum backdriving torque for all tested conditions. The minimum backdriving torque was 0.320 Nm for the unpowered system and 0.268 and 0.050 Nm with the 1DOF PID and 2DOF PID controllers, respectively. Thus, the 2DOF PID reduced the minimum backdriving torque by 81% compared to the 1DOF PID controller.

The measured exoskeleton joint torque and velocity data were processed offline with the System Identification Toolbox in MATLAB, using a two-pole one–zero model. For the unpowered system, the system identification resulted in an output impedance with equivalent inertia of 1.35 kg m^2^ and equivalent damping of 0.509 Nms/rad (Figure 4d). For the controlled system using the 1DOF PID, the identification produced equivalent inertia of 0.817 kg m^2^ and equivalent damping of 0.742 Nms/rad (Figure 4d). Finally, with the 2DOF PID controller, the equivalent inertia was 0.799 kg m^2,^ and the equivalent damping was 0.102 Nms/rad (Figure 4d). As expected, both PID controllers substantially reduced the output joint impedance compared to the unpowered case. However, the 2DOF PID controller provided substantial reductions of the output impedance by decreasing the equivalent damping by 86% compared to the 1DOF PID.

## 5. Human experiments

Three healthy subjects participated in the experiments (two males and one female, 28.0 ± 1.70 years old, 179 ± 7.50 cm tall, and 73.3 ± 5.77 kg). The experimental protocol was approved by the University of Utah Institutional Review Board. Written informed consent was provided by the subjects before the experiment took place. The subjects consented in writing to the publication of pictures and videos of the experiments.

**Figure 5.**
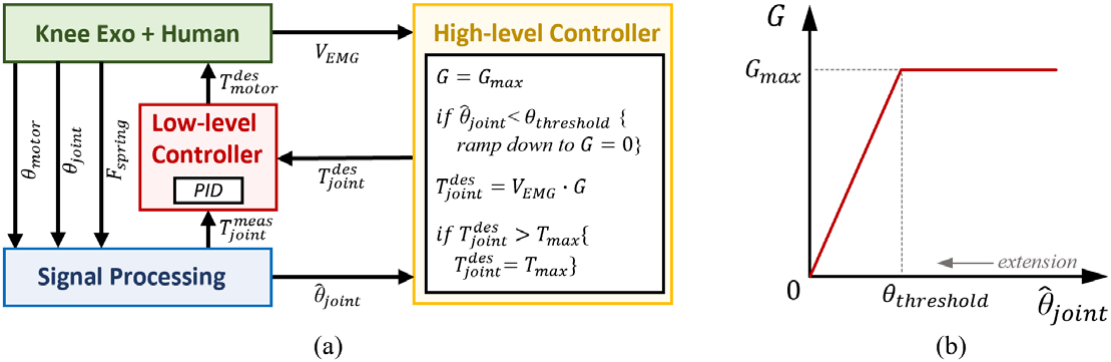
(a) Block diagram of the control and signal processing systems. At the high level, a proportional EMG controller defines the desired knee torque. At the low level, a closed-loop torque controller defines the desired motor current that is then imposed using a motor driver. (b) Relationship between the EMG gain 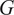 and 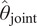 , as well as 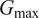 and 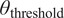 . In (b), the zero value of 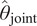 corresponds to full knee extension.

The exoskeleton aids the user based on the algorithm shown in Figure 5. The high-level controller uses the activation of the Vastus Medialis on the exoskeleton side to define the desired extension knee torque during stair ascents (Figure 5; Sarkisian et al., 2022). We chose Vastus Medialis for high-level control because it is a monoarticular knee extensor, unlike Rectus Femoris, which is a biarticular muscle spanning both the knee and hip joints. We also chose the Vastus medialis because it is easy to visually locate to achieve a correct EMG sensor placement. During the push-up phase, the EMG signal coming from the Vastus Medialis is multiplied by a proportional gain (*G = G*_max_) to define the desired knee extension torque. The EMG signals were rectified and filtered online by the commercially available sensor itself. The sensor provides the EMG envelope as an analog output, which is acquired by an on-board analog-digital converter within the embedded electronics at 2 kHz. The EMG signals were normalized by dividing the raw signal by the peak value of the EMG signal during the Transparent condition for each controller. The desired torque saturates at the maximum value ( 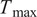 ) which was set to 0.5 Nm/kg to account for the subject’s body mass. As the knee extends and the knee joint angle gets below a set threshold 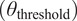 , the proportional gain 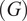 ramps down to zero. The controller parameters 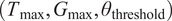 were adjusted by the experimenter through a graphical user interface. The proposed EMG controller was active during both the stance and the swing phases of the stair ascent.

**Figure 6.**
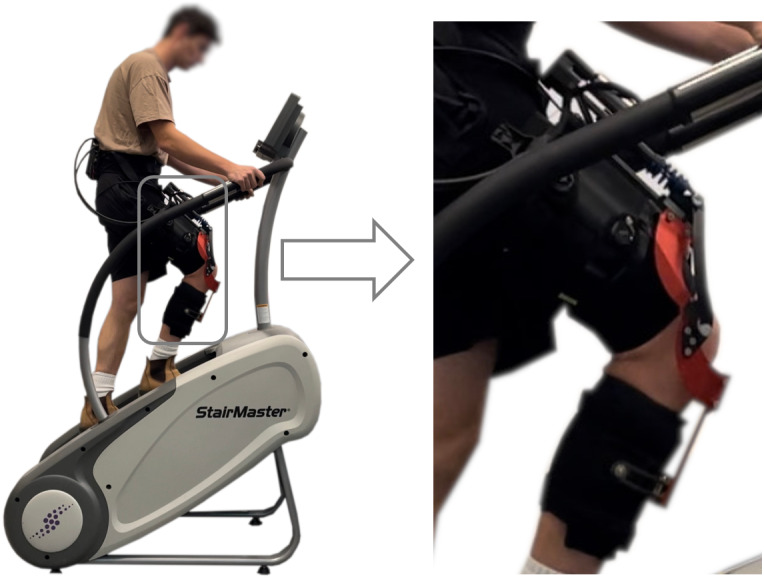
Experimental setup. The subject is wearing the exoskeleton and performing assisted stairs ascent. On the right, an enlarged view of the exoskeleton is shown.

Before the subjects donned the exoskeleton, we placed a single EMG electrode (13E200, Ottobock, Duderstadt, Germany) on their Vastus Medialis muscle, following SENIAM guidelines (SENIAM, n.d.). After donning the exoskeleton, the subjects climbed stairs on a StairMaster machine (Figure 6, right) at the fixed pace of 54 steps/min in zero-torque mode (i.e., transparent mode) and in assistive mode using the proposed EMG controller (Figure 2) (see Supplementary Material). The StairMaster machine has steps with a height of 15 cm (about 6 inches) and depth of 23 cm (about 9 inches). Each subject completed the stair climbing task with the 1DOF PID low-level controller and then with the 2DOF PID low-level controller. For all assisted conditions and all subjects, the peak of the assistive torque was set to 0.5 Nm/kg. The values of *G*_max_ were chosen experimentally to reach the desired level of peak torque. During the familiarization period, the experimenter gradually increased the value of *G*_max_ until the peak exoskeleton joint torque was reached consistently. The resulting values of *G*_max_ were 15, 8, and 8 for S01, S02, and S03, respectively. The values of *T*_max_ were chosen based on subjects’ body mass and were 35, 40, and 35 Nm for S01, S02, and S03, respectively.

**Figure 7.**
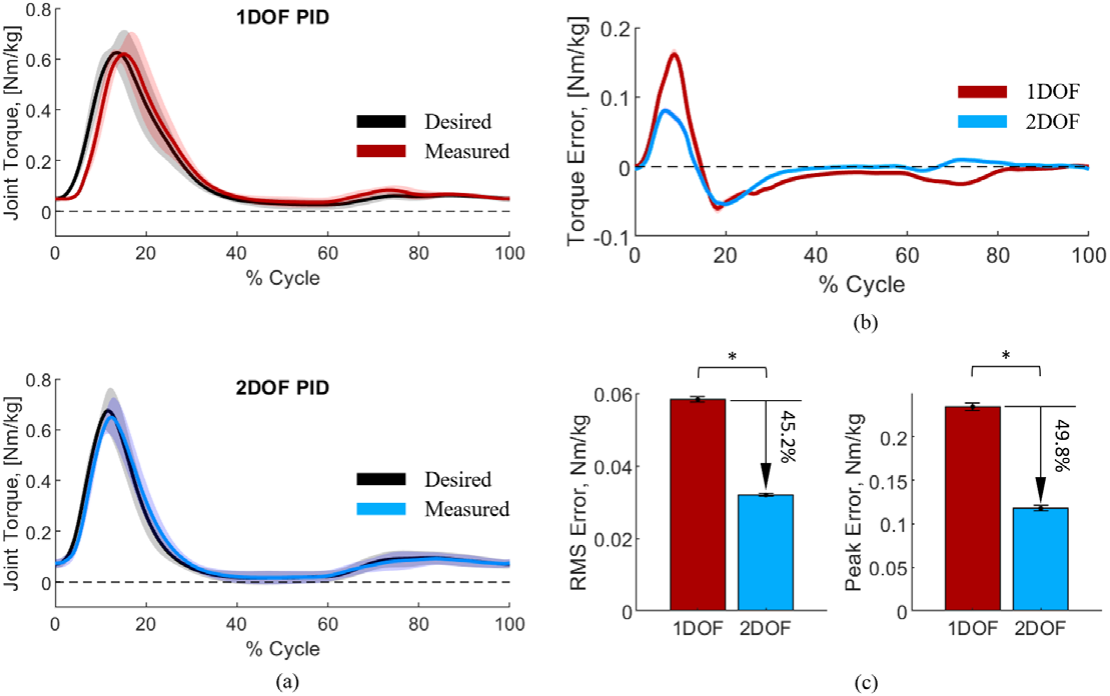
(a) Desired versus measured exoskeleton joint torque during assisted stair ascent. The data were normalized by subjects’ respective body mass. (b) Torque tracking error. (c) The RMS error and the peak error of the torque tracking. All data were averaged between individual stair gait cycles. The solid lines represent the mean, and the shaded regions and error bars represent the standard error.

We assessed the performance of the 1DOF PID and 2DOF PID controllers by quantifying the difference between the desired joint torque and the measured joint torque of the exoskeleton during the assisted stair-climbing experiments (Figure 7a). Specifically, we calculated the peak and the RMS error between the mass-normalized measured and mass-normalized desired torque (Figure 7b). The RMS error decreased from 0.0583 Nm/kg with the 1DOF PID controller to 0.0320 Nm/kg with the 2DOF PID controller. Additionally, the peak error decreased from 0.234 Nm/kg with the 1DOF PID to 0.118 Nm/kg with the 2DOF PID controllers. Thus, 2DOF PID reduced the RMS error and the peak error by 45.2 and 49.8%, respectively (Figure 7c). To statistically confirm these results, we conducted paired *t*-tests using the 1DOF and 2DOF controllers as independent variables and the RMS and peak errors as dependent variables, with correction for multiple comparisons. The results show statistical significance between the two low-level controller conditions (*p* < .00001).

**Figure 8.**
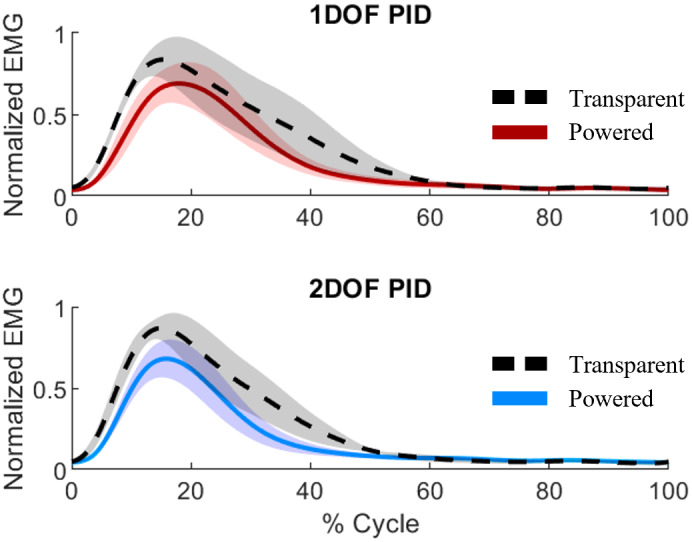
Data recorded by the EMG sensor. The data were first normalized by the peak value of the Transparent mode data for each condition and then averaged across subjects. Solid lines represent the mean, and the shaded regions represent the standard error.

We assessed the performance of the proposed high-level controller by comparing the EMG signals during the stairs climbing in transparent mode and in assistive mode (Figure 8). With the 1DOF PID controller, the peak EMG signals were 0.836 and 0.689 during the transparent and assistive modes, respectively. Thus, there was a 17.5% reduction in the EMG peak. With the 2DOF PID controller, the peak EMG signals were 0.869 and 0.680 during the transparent and assistive modes, respectively. This difference corresponds to a 21.6% reduction. Thus, both the 1DOF PID and 2DOF PID controllers lead to EMG reductions, although there does not seem to be a significant difference between the 1DOF PID and the 2DOF PID.

## 6. Discussion

### 6.1. Actuator design

Actuators are an essential part of powered exoskeletons and require a high level of output force while maintaining a low weight (Table 1). The proposed actuator’s mass is 0.725 kg, and it can produce up to 3,360 N of intermittent force and 170 W of mechanical power, resulting in a force density of 4,634 N/kg and power density of 235 W/kg. To the best of our knowledge, the linear SEA with the highest force density in the field achieves 2,397 N/kg and power density of 94 W/kg (Paine et al., 2014). Notably, both our SEA and the one presented in Paine et al. (2014) use the same motor. However, the difference in maximum intermittent force can be attributed to different motor driver capabilities. Thus, our SEA achieves almost twice the highest force and power density we could find in the literature (i.e., 2,397 N/kg and 94 W/kg (Paine et al., 2014).

**Table 1.** Actuator performance metrics

Mass	725 g
Size (contracted)	195 × 55 mm
Stroke	65 mm
Rated power output	170 W
Max continuous force	892 N
Peak force	3,360 N
Max speed	185 mm/s
Max efficiency	89%
Bandwidth	29.4 Hz
Force sensing resolution	11.0–15.2 mN

Our SEA uses a single coil spring with a tightly integrated coil-capturing system for both tension and compression. In contrast, the SEA shown in Paine et al. (2014) uses two springs, one engaging in tension and the other in compression. By using a single spring for both compression and extension, we eliminated the need for an additional spring, saving weight. Moreover, in our SEA, we use a method of attaching the spring to machined aluminum parts which reduces the number of components, including linear bearings. The proposed spring capturing mechanism is obtained by threading the open end of the spring into a machined channel that matches the pitch, diameter, and wire thickness of the spring (Figure 1a). In contrast, the SEA proposed in Paine et al. (2014) uses two opposing coil springs with two linear guides and an intermediate part for mounting. Thus, the higher force density of our SEA is mainly the result of a lower actuator mass, which is allowed by the proposed spring-capturing mechanism.

The location of the spring has a major impact on the performance of a SEA. In our SEA, the spring is located between the transmission system and the output load, which enables direct measurement of the output force. Conversely, the design in Paine et al. (2014) uses a reaction force sensing design, where the elastic element is placed between the ground and the motor. Although a reaction force sensing SEA can lead to a more compact design, it requires an accurate model of the plant to estimate the output force. If obtaining an accurate plant is a problem, then loadcells become necessary. The proposed SEA is designed to provide accurate torque control without requiring a model of the actuator.

### 6.2. Benchtop experiments

The spring characterization experiments show that the spring assembly is highly linear and that it has different stiffnesses in compression and extension. This result is due to the design of the proposed spring capturing system, which makes the effective spring length longer when the spring is in tension. To account for this difference, our low-level controller uses a function that outputs the correct stiffness values based on the sign of the spring deformation. The high linearity of the spring is key to achieving accurate closed-loop torque control. The torque step response for the 1DOF PID and 2DOF PID controllers are almost identical (Figure 4b). The only small but visible differences are in the overshoot (7.60%) and settling time (3.80%). In contrast, the two closed-loop torque controllers show completely different output impedance (Figure 4c). Interestingly, the equivalent inertias for the two controllers are comparable (~2% difference), but the equivalent damping is quite different (~86% difference). The big difference in the equivalent damping can be explained by the higher *K_P_*, *K_D_, and K_I_* gain action of the 2DOF PID controller, noting that the *b* and *c* weights only affect the reference value, which is zero for the output impedance test. This result shows for the first time the benefits of a 2DOF PID controller applied to SEAs. Tuning the closed-loop torque controller for SEAs is challenging due to potentially competing requirements on set-point following a disturbance rejection. The benchtop results strongly suggest that 2DOF PID control should be used in SEAs to reduce output impedance (i.e., improve backdrivability) without altering the set-point following characteristics, thus improving the performance of the device.

### 6.3. Human experiments

Our human experiments provide an assessment of closed-loop controller performance when the set-point and output joint position are changing at the same time. With the proposed EMG-based controller, the changes in set-point and output joint position happen dynamically, creating a mix of high-torque and high-speed requirements that need to be satisfied by the closed-loop controller. Our results show that the 2DOF controller reduces the torque RMS error and peak error by 45.2 and 49.8%, respectively (Figure 7c). Thus, the 2DOF PID controller provides substantially better torque tracking than a 1DOF PID controller. This study strongly supports the use of a 2DOF PID controller for powered exoskeletons driven by SEAs.

The comparison between transparent mode and assisted mode shows that the proposed proportional EMG controller can effectively reduce muscle effort compared to performing the same task in transparent mode. Interestingly, the 2DOF PID provides a slightly larger reduction of the peak EMG than the 1DOF PID controller. This result could be attributed to the better torque tracking performance of the 2DOF PID controller. However, more experiments are necessary to confirm this result.

### 6.4. Limitations

The main limitation of the proposed SEA design is its length. Because the elastic element is placed between the transmission and the load, it increases the length of the overall actuator without increasing the linear range of motion, which is a critical design parameter. This limitation could be addressed by increasing the diameter of the spring so that the ballscrew can move inside the spring, similar to the prosthesis described in Tran et al. (2022). However, this would require relocating the motor, which will result in an increase in the size of the actuator in another dimension. The length of the proposed SEA did not present any issues in the proposed powered knee exoskeleton. However, it may be a limitation for smaller wearable devices.

The step responses during the benchtop testing stage showed a 36.5% overshoot. This overshoot could be theoretically reduced by increasing the derivative gain. However, there are practical limits to the value of the derivative gain due to the noise in the online derivative. This issue can be partly mitigated by adding a filter at the cost of a resulting delay, which may cause instability. The human experiments show that the measured torque did not overshoot the torque set point during the tested assistive task. However, it is possible that a different tuning of the controller could result in higher accuracy of the closed-loop torque control.

Another limitation of this study is that we recruited only three able-bodied young subjects for the human experiments. Due to the smaller number of participants, it is unknown whether the observed EMG reductions are statistically significant. Experiments with a broader population are necessary to assess whether the results of this study generalize to a larger population.

## 7. Conclusion

In this study, we present a high-force density SEA with a 2DOF PID low-level controller for powered exoskeletons. To the best of our knowledge, this SEA has the highest force density in the field, primarily due to a lighter spring assembly that uses a single coil spring. Benchtop experiments show that the proposed spring assembly provides high linearity in both tension and compression. For the first time in a powered exoskeleton, we implement a 2DOF PID torque controller, which reduces output impedance (improves transparency) without requiring a model of the system or complex filters. Human experiments with a powered knee exoskeleton show that the 2DOF PID controller outperforms a common 1DOF PID controller during stair climbing by improving the accuracy of the torque controller. This study strongly supports the use of high-force density SEAs in combination with 2DOF PID control for powered exoskeletons.

## Supporting information

Sarkisian et al. supplementary material Sarkisian et al. supplementary material

## Data Availability

Data can be made available to interested researchers upon request by email to the corresponding author.

## Supplementary material

The supplementary material for this article can be found at https://doi.org/10.1017/wtc.2023.20.
